# No evidence of lymphatic filariasis transmission in Bamako urban setting after three mass drug administration rounds

**DOI:** 10.1007/s00436-022-07648-8

**Published:** 2022-09-06

**Authors:** Yaya Ibrahim Coulibaly, Moussa Sangare, Housseini Dolo, Lamine Soumaoro, Siaka Yamoussa Coulibaly, Ilo Dicko, Abdoul Fatao Diabaté, Lamine Diarra, Michel Emmanuel Coulibaly, Salif Seriba Doumbia, Abdallah Amadou Diallo, Massitan Dembele, Benjamin G. Koudou, Moses John Bockarie, Louise A. Kelly-Hope, Amy D. Klion, Thomas B. Nutman

**Affiliations:** 1 Mali - International Center of Excellence in Research (ICER-Mali), University of Sciences, Techniques and Technologies of Bamako, Bamako, Mali; 2 Dermatology Hospital of Bamako, Bamako, Mali; 3 Interdisciplinary School of Health Sciences | Faculty of Health Sciences, University of Ottawa, Ottawa, ON K1N 6N5 Canada; 4 National Lymphatic Filariasis Elimination Program, Ministry of Health and Public Hygiene, Bamako, Mali; 5 Centre Suisse de Recherche Scientifiques en Côte d’Ivoire, 01 BP 1303 Abidjan 01, Abidjan, Côte d’Ivoire; 6 UFR Science de la Nature, Université Nangui Abrogoua, 02 BP 801 Abidjan 01, Abidjan, Côte d’Ivoire; 7 School of Community Health Sciences, Njala University, Bo, Sierra Leone; 8 Centre for Neglected Tropical Diseases, Department of Tropical Disease Biology, Liverpool School of Tropical Medicine, Liverpool, UK; 9 Institute of Infection, Veterinary and Ecological Sciences, University of Liverpool, Liverpool, UK; 10 Laboratory of Parasitic Diseases, National Institute of Allergy and Infectious Diseases, National Institutes of Health, Bethesda, MD USA

**Keywords:** Lymphatic filariasis, Mass drug administration, Vector collection methods, *Anopheles gambiae* complex, Sudan savannah area, Mali

## Abstract

Lymphatic filariasis (LF) elimination activities started in Mali in 2005 in the most endemic areas and reached countrywide coverage in 2009. In 2004, the district of Bamako was endemic for LF with a prevalence of 1.5%. The current study was designed to determine LF endemicity level in the urban area of Bamako after three rounds of ivermectin and albendazole mass drug administration (MDA). A cross-sectional study was conducted in 2011 in Bamako city, consisting of human prevalence and entomological surveys. Volunteers aged 14 years and above were invited to participate and tested for evidence of *Wuchereria bancrofti* using night time blood thick smear microfilarial count and blood spots for LF antibodies using the SD BIOLINE Oncho/LF IgG4 Biplex rapid test (Ov16/Wb123). Mosquitoes were collected using CDC light and gravid traps and tested using molecular methods. Poolscreen software v2.0 was used to estimate vector transmission potential. Of the 899 volunteers, one (0.11%) was found to be positive for LF using the Oncho/LF IgG4 Biplex rapid test, and none was found to have *Wuchereria bancrofti* microfilariae. No mosquitoes were found infected among 6174 *Culex* spp. (85.2%), 16 *Anopheles gambiae s.l.* (*An. gambiae s.l.*) (0.2%), 26 *Aedes* spp. (0.4%), 858 Ceratopogonidae (11.8%) and 170 other insects not identified (2.3%) tested. Our data indicate that there was no active LF transmission in the low prevalence urban district of Bamako after three MDA rounds. These data helped the National LF programme move forward towards the elimination goal.

## Introduction

Of the five genera of mosquitoes that transmit lymphatic filariasis (LF), *Culex* (*Cx.*) is the most common worldwide and represents, through the *Cx. pipiens* complex (especially *Cx. quinquefasciatus*), the principal vector of nocturnal periodic *Wuchereria bancrofti* (*W. bancrofti*) in urban areas of Asia, eastern Africa, the West Indies, South America and Micronesia (Bockarie et al. 2009). The high density of *Cx. quinquefasciatus* in urban areas is due to the high frequency of the specific types of breeding sites this species prefers, such as different types of stagnant, often polluted, water (Castro et al. 2010). This also leads to their persistence even in the dry season. Based solely on the vector-parasite relationship, *Culex* species (*Culex* spp.) are potentially more competent for LF transmission than anopheline mosquitoes, especially when the microfilarial load is low, as would be the case following mass drug administration (MDA) (Curtis et al. 1983; Curtis et al. 1981). In West Africa, including Mali (Touré 1979), anopheline species are less abundant in urban areas than *Culex* spp. but are the main vectors of LF (de Souza et al. 2012). This may be due to decreased susceptibility of *Culex* spp. to West African strains of *W. bancrofti* (Cano et al. 2014), as compared to strains from India, Sri Lanka (Kuhlow and Zielke 1978) and Tanzania (Curtis et al. 1981).

In Mali, LF elimination activities started in 2005 in the most endemic areas and scaled up to reach 100% geographic coverage in 2009 (Dembélé et al. 2012). In the urban district of Bamako, low prevalence (1.5%) was found among the 599 people tested (about 100 persons per locality in six localities) using the immunochromatographic card test (ICT); only nine people were positive (0 positive in 2 localities, 1 positive in 2 localities, and 5 and 2 positives respectively in 2 localities) (National LF Elimination Program 2004 LF mapping report). Localities with positive subjects were in the peripheral peri-urban areas of the city posing the necessity to consider urban and peri-urban components of large cities such as Bamako as separate implementation units (Koudou et al. 2018). Three rounds of MDA were conducted in Bamako prior to the initiation of the current study with treatment coverage rates of 77%, 100% and 100% in 2008, 2009 and 2010 (Adams et al. 2018), respectively. The aim of this study was to assess the prevalence in a low endemicity urban setting after three rounds of MDA.

## Methods

### Study design and sites

The study was conducted in Bamako, the capital city of Mali in West Africa. It is the most populated of the 63 districts of the country with an estimated population of 1,810,000 inhabitants in 2009. The city is located in the Sudan savannah area, covers an area of 1420 km^2^. It has a tropical wet and dry climate with the Niger River running through its centre. Cross-sectional surveys of human prevalence were conducted across eight quartiers of the city, which included six programmatic sentinel sites in six quartiers plus two additional sites with similar geographical characteristics (Bakaribougou, Bozola, Dialakorodji, Faladiè, Niamakoro, Sabalibougou, Sirakoro dounfing and Taliko).

### Human prevalence

In March–April 2011, blood samples from volunteers in the eight quarters were collected. First, the head of each quartier as well as the local leaders were convened to explain the purpose of the study and obtain community consent for both the entomological and parasitological components of the study. The research team worked with the local health workers through the whole process. Volunteers aged 14 years and above who were permanent residents in the selected localities were invited to participate in the study. The volunteers, after signing an informed consent form if aged 18 and above, or an assent form if < 18 years in addition to the consent form signed by a tutor, underwent a brief health history interview focused on LF. Depending on the pathology detected by the physician, advice was provided as well as assistance or free medicines if needed and available with the research team.

Blood samples were collected using finger prick on site for three calibrated 20-µL blood films on three different glass slides between 10 pm and 2 am as well as three blood spots of 20 µL each on Whatman® filter papers. The following day, the slides were dried on site and sent to the laboratory for 5% Giemsa staining and reading by experienced stereomicroscopists. The dried blood spots (DBS) were stored in individual envelopes at − 80 °C with a desiccant (silica gel) for subsequent *W. bancrofti* Wb123 antibody detection using the SD BIOLINE Oncho/LF IgG4 Biplex rapid test (Ov16/Wb123) (Steel et al. 2015). At the time of testing, the DBS samples were thawed and a 6-mm disc punched out and placed in a 96-well elution plate. Elution buffer (100 μL) was added to each well and pipetted up and down to mix. The plate was covered and incubated overnight (12–24 h) at 2–8 °C. The following day, the sample was mixed again with a pipet prior to the addition of a 10 µL sample of DBS eluate to the appropriate wells of the test strips. Test results were read after 30 min (Steel et al. 2015).

### Entomological data

Vector collections were conducted in October 2011. During the day of collection in each quartier, six CDC light traps (indoor) and six CDC gravid traps (outdoor) were used from 6 pm to 6 am. Each light trap was in a volunteer’s room and operated after removing all the other sources of light. It was suspended at about 1.3 m above the floor of the room, close to the occupant who used a bednet. At each of the eight collection sites, one light trap and one gravid trap were operated 100 m apart.

### Entomological processing

Mosquitoes were sorted by morphology into distinct species (*Culex* spp., *An. gambiae s.l.*, *Anopheles funestus s.l.*, other *Anopheles* species and *Aedes* spp.) and stored in pools of one to 30 according to the collection method and the quartier in 1-mL Nunc® Tubes containing absolute alcohol (Dahan-Moss et al. 2020). The next day, they were stored at room temperature in the laboratory before the PCR processing to detect *W. bancrofti* DNA. The PCR technique used was previously described by Rao et al. in 2006 (Rao et al. 2006).

### Data management and analysis

Vector infection likelihood and the related 95% confidence intervals were estimated using Poolscreen v2.0 software (Katholi and Unnasch 2006). Collected data were analysed using SPSS version 25 (SPSS Inc., Chicago, IL) and GraphPad Prism version 5 (GraphPad Software, La Jolla, CA) softwares. For proportion comparisons, the chi^2^ test or the Fisher’s exact test was used as appropriate.

## Results

### Parasitological data

The number of subjects enrolled in the eight localities ranged from 81 (Faladie) to 207 (Dialakorodji). In total, 1002 volunteers were enrolled, and women (66.3%) and people aged 14–24 years (42%) made up the majority (Table 1). All 1002 night time blood thick smears were negative for *W. bancrofti*. Of the 1002 volunteers, 899 were tested using Oncho/LF IgG4 Biplex rapid test (Ov16/Wb123) with only one volunteer found positive for LF (Wb123) in Dialakorodji quartier (0.11% (1/899) Table 2).

**Table 1:** Characteristics of the study population

Localities	Total enrolled	Gender		Age groups (years)					
		Women (%)	Men (%)	14–24	25–34	35–44	45–54	55–64	≥ 65
Bakaribougou	149	63.1	36.9	56	22	9.4	6	2.7	4
Bozola	141	85.1	14.9	57	16	11	8.5	3.6	4.3
Dialakorodji	207	70.1	29.9	38	17	17	14	8.7	5.3
Faladie	81	43.2	56.8	41	19	17	11	9.9	2.5
Niamakoro	88	80.7	19.3	49	14	23	8	4.6	2.3
Sabalibougou	100	54	46	35	23	20	12	6	4
Sirakoro dounfing	142	68.3	31.7	25	17	11	21	13	13
Taliko	94	51.1	48.9	33	20	13	11	11	13
Total	1002	66.3	33.7	42	18	15	12	7.3	6.2

**Table 2:** *Wuchereria bancrofti* infections prevalence variations in the eight study localities of Bamako using the Biplex on filter paper dried blood sample

Localities	Total enrolled	Number tested	Positive	
			Wb123	%
Bakaribougou	149	148	0	0
Bozola	141	65	0	0
Dialakorodji	207	207	1	0.48
Faladie	81	81	0	0
Niamakoro	88	88	0	0
Sabalibougou	100	74	0	0
Sirakoro dounfing	142	142	0	0
Taliko	94	94	0	0
Total	1002	899	1	0.11

### Entomological data

A total of 6174 *Culex* spp. (85.2%), 16 *An. gambiae s.l.* (0.2%), 26 *Aedes* spp. (0.4%), 858 Ceratopogonidae (11.8%) and 170 other insects not identified (2.3%) were collected. The 6174 *Culex* spp. were pooled into 1 to 30 specimens per pool to make the 252 pools that were tested. Two additional pools made with the 16 *An. gambiae s.l.* and the 26 *Aedes* spp. were also tested. No infected pool was identified using PCR (Table 3).

**Table 3:** Number of flying insects collected per species in the seven visited localities of Bamako in October 2011

Localities	*Culex* spp.		*An. gambiae s.l*		*An. funestus*		*Aedes* spp.		Ceratopogonidae		Other		Total
	*N*	%	*N*	%	*N*	%	*N*	%	*N*	%	*N*	%	*N*
Faladie	1318	95.6	2	0.1	0	0	4	0.3	34	2.5	20	1.5	1378
Bakaribougou	1235	95.1	2	0.2	0	0	0	0	42	3.2	19	1.5	1298
Dialakorodji	572	91.1	4	0.7	0	0	7	1.1	16	2.5	29	4.6	628
Niamakoro	710	85.7	1	0.1	0	0	6	0.7	90	10.9	21	2.5	828
Sabalibougou	1309	75.9	0	0	0	0	2	0.1	400	23.2	14	0.8	1725
Sirakoro	274	47.2	6	1	0	0	4	0.7	254	43.7	43	7.4	581
Taliko	756	93.8	1	0.1	0	0	3	0.4	22	2.7	24	3	806
Total	6174	85.2	16	0.2	0	0	26	0.4	858	11.8	170	2.3	7244

*Sirakoro*, Sirakoro dounfing; *spp.*, species

## Discussion

Our data provide evidence for a lack of LF transmission in Bamako, which is in line with what has been found in other large West African cities (de Souza et al. 2014). Moreover, our results suggest that *Culex* mosquitoes are the most frequent mosquitoes in Bamako, which are not known as vectors of LF in Mali (Coulibaly et al. 2016; Coulibaly et al. 2006; Coulibaly et al. 2015). Low baseline transmission combined with the three MDA rounds completed before the initiation of the current study likely explains these results. Although early assessments suggested the presence of LF transmission in Bamako, this may have been due to false positive results obtained with certain batches of the ICT used in Mali at that time (Chu et al. 2013; Joseph et al. 2011).

Some studies have shown through laboratory experiments that both vector mosquitoes and non-competent vector species may contain parasite DNA (Cook et al. 2017; Erickson et al. 2009; Fischer et al. 2007; Mukabana et al. 2002). Nonetheless, positive parasitic DNA test results in mosquitoes does not provide definitive evidence that transmission is occurring in the study area. The human data strongly suggest that LF is not a public health problem in Bamako and that active transmission is not occurring. Although *An. gambiae s.l.* abundance was relatively low in the study site due to the fact that the study was conducted during the dry season (Sissoko et al. 2015), the total number of mosquitoes collected was substantial and would allow detection of infection rates as low as one infected female out of 1000 tested.

We processed 6174 *Culex* spp. with no infection identified. Such a low *W. bancrofti* microfilarial prevalence could be expected after three MDA in endemic areas if initial prevalence were low. Similar results have been reported by other studies around the world (Farid et al. 2007; Goodman et al. 2003; Mehta et al. 2018). In Bamako, given the limitation pattern that characterises the *Culex* spp. and increases its ability to take up microfilariae and bring them to the infective stage even if the microfilarial load is very low (Pichon 2002), results from such number of vectors is strongly suggestive of a lack of transmission. This is due to the lack or very rare mosquito-infected human host contact as demonstrated by the current xenomonitoring findings. It has been reported that even mosquitoes that feed on a microfilarial carrier can be detected as positive because of *W. bancrofti* DNA in the ingested blood (Rao et al. 2006). Moreover, the absence of microfilariae in the tested samples highlights the non-availability or rarity of an infection reservoir for the local mosquitoes to sustain transmission in Bamako. Surveillance should be continued as in any endemic or at risk of transmission area in order to detect early resurgence or appearance of LF (Chu et al. 2013).

After our study in 2011, additional MDA rounds were conducted in 2011, 2012, 2013, 2015 and 2016, followed by a series of transmission assessment surveys (TAS). TAS 1 was conducted in 2016 with 0 positive out of 3471 children tested; TAS 2 in 2018 with 0 positive out of 3430 children tested and finally TAS 3 in 2022 with 1 positive child out of 7905 children tested. Based on these recent results from the various evaluations, we can say with confidence that LF transmission is interrupted in Bamako. All of the other evaluation units in Mali (*n* = 19) also underwent the three required TAS. These results are available from the National Program for the Elimination of Lymphatic Filariasis in Mali upon request and have not been published yet. As a result of the TAS data, MDA was stopped in all evaluation units in Mali in 2016.

## Conclusion

Based on the collected data, three rounds of MDA were sufficient to interrupt transmission in Bamako, the urban centre of Mali.

## Data Availability

The data that support the findings of this study are available from the corresponding author (Moussa Sangare, mbsangare@icermali.org), upon reasonable request.

## Abbreviations

CDC: United States Center for Disease Control and Prevention
DNA: Deoxyribonucleic acid
GPELF: The Global Programme to Eliminate Lymphatic Filariasis
ICT: Immunochromatographic test
LF: Lymphatic filariasis
MDA: Mass drug administration
PCR: Polymerase chain reaction
