# Correction to: No evidence of lymphatic filariasis transmission in Bamako urban setting after three mass drug administration rounds

**DOI:** 10.1007/s00436-022-07718-x

**Published:** 2022-11-10

**Authors:** Yaya Ibrahim Coulibaly, Moussa Sangare, Housseini Dolo, Lamine Soumaoro, Siaka Yamoussa Coulibaly, Ilo Dicko, Abdoul Fatao Diabaté, Lamine Diarra, Michel Emmanuel Coulibaly, Salif Seriba Doumbia, Abdallah Amadou Diallo, Massitan Dembele, Benjamin G. Koudou, Moses John Bockarie, Louise A. Kelly‑Hope, Amy D. Klion, Thomas B. Nutman

**Affiliations:** 1 Mali ‑ International Center of Excellence in Research (ICER‑Mali), University of Sciences, Techniques and Technologies of Bamako, Bamako, Mali; 2 Dermatology Hospital of Bamako, Bamako, Mali; 3 Interdisciplinary School of Health Sciences, Faculty of Health Sciences, University of Ottawa, Ottawa, ON K1N 6N5 Canada; 4 National Lymphatic Filariasis Elimination Program, Ministry of Health and Public Hygiene, Bamako, Mali; 5 Centre Suisse de Recherche Scientifiques en Côte d’Ivoire, 01 BP 1303 Abidjan 01 Abidjan, Côte d’Ivoire; 6 UFR Science de la Nature, Université Nangui Abrogoua, 02 BP 801 Abidjan 01 Abidjan, Côte d’Ivoire; 7 School of Community Health Sciences, Njala University, Bo, Sierra Leone; 8 Centre for Neglected Tropical Diseases, Department of Tropical Disease Biology, Liverpool School of Tropical Medicine, Liverpool, UK; 9 Institute of Infection, Veterinary and Ecological Sciences, University of Liverpool, Liverpool, UK; 10 Laboratory of Parasitic Diseases, National Institute of Allergy and Infectious Diseases, National Institutes of Health, Bethesda, MD USA

**Correction to: Parasitology Research (2022) 121:3243–3248**

**https://doi.org/10.1007/s00436-022-07648-8**

The article “No evidence of lymphatic filariasis transmission in Bamako urban setting after three mass drug administration rounds”, written by Yaya Ibrahim Coulibaly, Moussa Sangare, Housseini Dolo, Lamine Soumaoro, Siaka Yamoussa Coulibaly, Ilo Dicko, Abdoul Fatao Diabaté, Lamine Diarra, Michel Emmanuel Coulibaly, Salif Seriba Doumbia, Abdallah Amadou Diallo, Massitan Dembele, Benjamin G. Koudou, Moses John Bockarie, Louise A. Kelly‑Hope, Amy D. Klion, and Thomas B. Nutman, was originally published online on September 6, 2022 with Open Access under a “Creative Commons Attribution (CC BY) licence 4.0”. After publication in volume 121, issue 11, page 3243–3248, the author(s) decided to cancel the Open Access. Therefore, the copyright of the article has been changed on October 30, 2022 to © The Author(s), under exclusive licence to Springer-Verlag GmbH Germany, part of Springer Nature 2022 with all rights reserved.

The original article has been corrected.

